# Finding Characteristics of Users in Sensory Information: From Activities to Personality Traits

**DOI:** 10.3390/s20051383

**Published:** 2020-03-03

**Authors:** Jaeryoung Lee, Nicholas Bastos

**Affiliations:** Department of Robotic Science and Technology, College of Engineering, Chubu University, Kasugai, Aichi 487-8501, Japan; nicholas.bastos@gmail.com

**Keywords:** sensor-based activity recognition, sensor-based habit assessment, personality traits, data clustering

## Abstract

The main objective of this work was to use information provided by a sensor-based activity recognition system to create a profile comprising user habits and link this information to the personality traits of users. User habits are represented by the sequence and duration of often observed daily life activities. Based on this information, we represented the user trials (sequence of activities) following a numerical method using Fourier series. The duration and sequence of the activities changed the phase and amplitude of the harmonics present in the Fourier representation. Each trial represented in this manner is called a behavioral spectrum. These data and the scores obtained from personality questionnaires were clustered separately and then an association was created between the clusters. The objective was to associate the activity-related features (sensor-based) and personality traits. The experimental results showed that for both young and elderly subjects, there is an association between the user personality traits and the manner in which they perform their activities. Moreover, the results obtained in this work show a promising method of assessing the personality traits of users based on their activities.

## 1. Introduction

Assessing the personality of an individual is not a simple task, owing to the complex nature of its definition. According to Allbert et al. [1], personality is the dynamic organization of those psycho-physical systems within an individual that are determined by behaviors and thoughts. Although this definition emphasizes the uniqueness of the psychological structures of the individuals, several studies over the years have attempted to identify patterns and traits shared between groups of people. One of the famous studies regarding the classification and identification of personalities is the work of Eysenck [2,3,4]. Eysenck proposed a theory of personality based on biological factors, arguing that individuals inherit a particular type of nervous system that affects their ability to learn and adapt to the environment. Based on a series of questionnaires and analyses via case studies, he observed that the personality of an individual could be classified according to some traits or factors grouped together under separate headings, called dimensions. He proposed three main dimensions, introversion/extroversion (E), neuroticism/stability (N) (second-order personality traits), and psychoticism/normality (P) (which are sometimes indicators of mental disorder). Based on the scores obtained for each dimension, the personality traits of an individual could be traced to an area in this three-dimensional space. This model is commonly known as the PEN model. Reducing personality traits of individuals to three dimensions is an extremely large dimensional reduction for such complex aspects of human characteristic. Based on the contribution of several researchers over the years, a similar method was developed. This method is referred to as the big five personality traits approach [5]; this is an expansion of the PEN model to include five different features observed in human behavior. Similar to the PEN model, personalities of individuals can be traced to a point or area in five-dimensional space according to the score of each feature. Moreover, some of the traits used in the PEN model can have direct relationships with some of the traits used in the big five traits approach.

### 1.1. Personality Trait Computation

As mentioned above, most studies refer to the big five model [6]; however, it is difficult to apply this model for identifying personality traits in computing applications, because of the complex dimension. Therefore, Dotti et al. [7] applied the following alternative personality categories: resilient, overcontrolled, and undercontrolled (ROU model) [8,9]. Our work used the big five model in this paper, but we calculated the association in the aspect of extroversion and neuroticism, and only neuroticism, which is similar to the ROU model.

Understanding these aspects regarding the personality traits of individuals has proved useful and effective in human–computer interactions [10]. Knowledge regarding user personality traits can lead to more satisfying interactions and higher acceptability levels between users and smart devices [11] or robots [12]. Predicting personality traits in computing applications is accomplished using either verbal or nonverbal cues [6]. Although some studies focused on extracting video and audio features from human–computer interactions [7,13], most of the recent work on personality trait identification using automatic computing processes focuses on analyzing text present in social media and electronic messages [14]. Based on text data found in the *Whatsapp* application and SMS, the work presented by [14] uses a classifier module that can relate specific words with characteristics for defining user personalities based on the PEN and big five trait models. In a similar fashion, from a dataset based on smartphone usage (call logs, SMS, app usage, etc), a supervised machine learning system (based on SVM) shows that several aggregated features can be indicators of the big five traits [15]. The main idea behind these techniques is to use a large number of social interactions (either application usage logs or direct processing of text conversations) to find features that can be related to specific traits in the dimensions of the PEN model or the big five personality trait approach [16,17,18]. Although these approaches have led to many findings on how personality traits can be predicted from smart phone usage [15], there are some concerns regarding user privacy and scope of these approaches; their scope is limited to virtual interactions, depending on which applications are popular among young users [19]. Furthermore, there are few computer-focused approaches using activity-related features to identify user personality traits [7]. Approaches that work with alternative features usually focus on identifying specific business-oriented aspects of user personalities.

In traditional studies, the personality traits are estimated using the responses provided in the questionnaires and a psychological approach is used; however, in several recent studies, the estimations were performed using mathematical approaches. As mentioned above, most of the studies utilized the footprints of the user in social network services or smart phone messages [14,15,16,17,18,19], whereas others used the data of the user behaviors, in particular, a vision data or multimodal human data [6,7,13]. The novelty of the approach lies in the fact that the duration and sequence of activities in daily life are considered to be the main parameters in the estimation of the personality traits of the users. This makes the process more cost-effective and computationally less intensive as compared to the case when vision or human modality data is used. Furthermore, data collection using a sensor-based system is convenient. Therefore, to the best of our knowledge, this is the first study that estimates the personality traits based on sensor-based information (excluding vision data) in the field of human-computer interaction.

### 1.2. Contributions and Paper Overview

The work presented in this paper is motivated by the daily life of a user in a smart home. This sensor-based information can be used to find the habits and personality traits of the users by analyzing the activities. Therefore, to identify the personality traits of users, a method that uses information obtained from the sequence and duration of user activities is presented. The two aforementioned parameters, sequence and duration of activities, are accessible from the database of a simple activity recognition system such as a low-cast and low-energy smart home [20]. For studies concerning activity recognition, Liu et al. proposed a sensor-based system with a learning method that can identify more complex activities of users [21,22,23]. They developed an unsupervised method to recognize user’s activities using the smartphones accelerometers [24]. Inertial sensors installed in a smartphone have also been used in another study to identify individual physical activities [25]. For more precise recognition, more complex frameworks have been developed that use mutimodal data to train a deep neural network [26]. Other studies used smart home datasets for feature representation along with the application of long short term memory recurrent neural network method [27]. Wang et al. summarized the studies of activity recognition systems regarding sensing modalities, types of sensor distributions, and learning models [28]. Notably, the method and features of activity recognition systems can range from extremely simple to complex depending on the purpose of information and the type of users. The activity recognition performed utilizes an extremely simple method and features [20] compared to those in other studies [28]. Groups of similar activities can be identified as habits that are presented in a numerical form (this habit system is described in Section 2). The frequency and number of activities indicated in a habit representation are found to be associated with a specific dimension in the personality trait classification. For example, the number of times a user switches between activities could be an indicator of low stability scores, a trait that can be referred to as neuroticism, which is one of the big five personality traits. Although there are limitations to the accuracy with which the personality traits can be identified using only the information obtained from habits and activities, such approaches reveal associations between user activities and habits along with the peak scores of personality traits.

**Figure 1:**
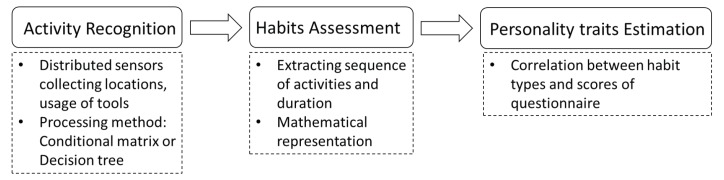
Novel approach to identify personality traits of humans based on their activities. The activity recognition system requires multiple distributed sensors for collecting information on locations and tool usage. Note: this system did not collect video data because of privacy concerns and an energy issue of the system hardware.

To determine habits from user activity information, we used the extended version of a previous method [29]. This method uses a novel approach to represent habits based on activity information from a mathematical point of view (see Figure 1). The activity recognition system provides information regarding the sequence and duration of the observed activities. Based on these parameters, the activity trials are represented in a numerical format using Fourier series representation. The output signals from different users are clustered into groups using the k-means algorithm, where each cluster corresponds to a potential habit. The concept of habits is restrictively linked to the interpretation of the activities from the view point of the activities of daily living; thus, only a small set of the most important activities is used in this stage.

The definition of habits used in this work is related to user activities; a habit is considered a combination of several patterns regarding how, where, and when the users accomplish their daily activities. This definition was chosen because the concept of habits is usually related to user activities [30,31]. In our developed method, a habit is defined according to the observed activity sequence and duration of each one, according to a specific time interval. In other words, habits represent a set of parameters found based on the recognized activities. The main objective of this work was to establish an association between user personality traits and activities, based on the proposed numerical representation of trials and habits using Fourier series. Moreover, an extended and detailed version of the numerical representation of habits provided in earlier studies was developed [29]. The results and method presented in this study can be used to improve the personality trait estimation by considering the association of personality traits with user activities using nonverbal cues and can be applied in human-robot interaction. For example, the interaction of robots with users can be significantly improved according to the mathematically represented habits or personality traits of users.

The remainder of the paper is organized as follows: in the Habit Assessment System section, the general idea behind the habit assessment system is explained, which is an extension of our previous work. In the same section, we explain mathematical details about the representation of each trial as a signal and how to cluster and associate that information to identify user habits. In the Correlation with User Personality Traits section, we present details about two different sets of experimental data used to extract the desired features and validate the proposed method. The correlations between user habits and personality traits are described in the Correlation Results between Personality Scores and Activity Series section; further discussions along with the conclusions are presented in the Discussion and Conclusion sections.

## 2. Habit Assessment System

This work was mainly carried out to express the correlation between user personality traits and habits. To achieve this objective, we expanded a previously developed system [29]. The method developed in this study involves representing user habits in a numerical format based on the sequence and duration of the observed activities. This numerical representation helps in extracting and analyzing certain features concerning user activities and habits. Because the concept of habits can be defined in several ways, it is important to clearly define the ones used in this study.

### 2.1. Habit Analysis

In our previous study, we developed a system for habit analysis [32]. The concept of habits itself can be quite abstract; it is defined as *something that you do often and regularly, sometimes without knowing that you are doing it*. This overall definition includes the basic concept of a habit being a pattern observed regarding the start and end time, for each activity in particular. For example, a user takes a nap from 12:00 (start time) to 13:00 (end time) almost every day. It is also possible to identify user habits from the basic definition of a habit, by observing the sequence followed by the user to perform simple activities. For example, the user always takes a nap for approximately an hour (one activity) after eating lunch (another activity). To identify the habits of users (based on a set of activities performed within a time interval). First, sensor information is collected to obtain a better understanding of how the user interacts with the environment. This information is used by the activity recognition system [33] that uses the state of the environment to recognize a specific set of the most important activities in the daily lives of users. The activity recognition system is described in a previous work [34]. Then, all the observed activities within a specific time interval (morning for example) are represented in a numerical format based on their occurrences (order of identification and duration). For example, if a user performs the activities (in order) of eating breakfast (for 30 min) and watching TV (for 1 h), then the sequence will be [1:(eating_breakfast);2:(watching_tv)], and the duration for each one will be [1:30_min;2:60_min]. Each activity name is associated with a unique ID, allowing precise numerical representation.

Based on these values, a mathematical representation of one trial is created. The mathematical representation used in this work is based on the Fourier series, where the above mentioned activity characteristics are used to create a periodic signal. The ID number of one activity has a direct correlation with one specific harmonic phase and the duration is related with the amplitude of the harmonic. The order in which activities are performed directly affects one specific harmonic, according to its respective number (skipping the first harmonic, which represents the average of the signal). For example, the first activity performed in an overall habit is always related to the second harmonic, the second activity with the third harmonic, and so on. The details regarding signal creation are explained later in this section. The signal obtained based on user activities is called the behavioral spectrum [32]. This signal represents one trial that could be one potential habit.

**Figure 2:**
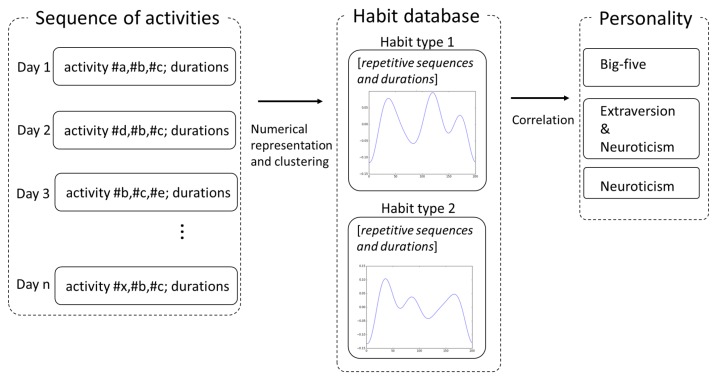
Estimation of personality traits from the sequences of activity data.

This numerical representation is clustered into groups where each one represents the category of an overall habit with similar behaviors (see Figure 2). For example, let us assume that a specific user often prays for a few minutes and then takes a shower during the night. Creating a numerical representation (behavior signal) based on these factors and grouping the data from several days of analysis (one week of analyzing what the user activities are during the night, for instance) we can create one cluster representing the habit of praying and then taking a shower. When unusual behavior is observed (praying after taking the shower), the numerical representation of that strange behavior will not fit in the same group (or cluster) created before; a warning is sent to the system stating that a behavior not representing an overall habit is identified.

Analyzing each of the habits associated with different clusters enables identification of some characteristics regarding how users perform their activities. For example, the behavioral spectrum associated with a high frequency represents a group of overall habits where the user usually performs more activities because the number of activities within in an overall habit is associated with the harmonic phase in the proposed method. This analysis could explain some aspects of the stability dimension in the PEN, big five, and ROU models. The frequency of the signal is directly related to the number and sequence of activities in a trial; thus, the frequency observed in a particular behavioral spectrum can be related to the stability score of a personality trait. The same idea can be applied to identify cultural characteristics, where a specific cultural background is associated with a specific group of habits.

### 2.2. Clustering Behavioral Spectrums to Find User Habits

Because the habits of users are found after analyzing the patterns observed in the user activities and behaviors, it is not possible to recognize user habits using information on only one instance. Instead, it is necessary to analyze user activities and behaviors across several days, where each pattern in their behavior represents a routine or habit. This is possible after grouping several behavioral spectrums to find similarities between the recorded data. These similarities represent the habits of the users. To identify these similarities, a clustering process for finding similarities in groups of data was used [35]. It attempts to group information in a population together based on similarity, but not oriented by a specific label, which are outputs values to which an algorithm maps data points. Labels were not used in the present work because the main objective was to find an association between the patterns obtained and the personality traits rather than developing a personality recognition system, which may be difficult to achieve using only activity information.

**Figure 3:**
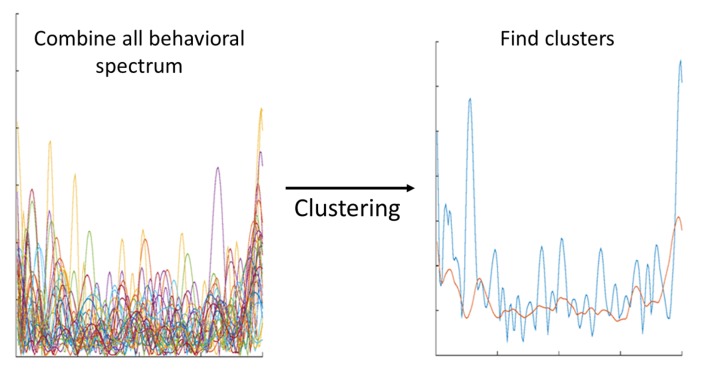
Example of the methodology used to identify habits from behavioral spectrum data.

As one of the objectives of this work involved comparing the results of the obtained clusters (activity information and personality trait scores), it was important to choose a clustering method that facilitated easy interpretation of the results. Therefore, the k-means method was used for clustering [35,36]. This method involves finding centroids in each data dimensionality that will represent one cluster of data in that specific dimension [36]. The clustering method used in this work is an extended version of the method used in a previous work [29]. The previous version had a high chance of transforming into the local minimum problem, which could lead to incorrect associations between habits and some trials. As explained before, the main purpose of the clustering method is to find similar patterns between several trials recorded across different days. Each pattern found can express a different habit of the user. An example of the clustering process is shown in Figure 3.

Because the clustering process starts at random points automatically selected by the algorithm, it is important that there are several interactions in order to find the one that will provide the best centroid positions [35]. The quality of each interaction is judged according to the percentage of variance found in the index vector presented by the k-means algorithm.

This vector correlates the observations with their respective associated clusters. A low variance means that the clustering interaction did not produce suitable centroid points that represent data generalization. On adding another cluster, more data will be represented by the newly added cluster, thus spreading the representation and increasing the percentage of variance explained by the total number of clusters. The percentage of variance is the ratio of the between-group variance to the total variance, also known as an F-test [35].

To determine which behavior corresponds to which cluster, it is necessary to calculate the mean average error (MAE) between all the clusters found and the input behavioral spectrum. The MAE with the lowest value corresponds to the habit to which that behavioral spectrum fits. Thus, when a new behavior is observed, it will fit into one of the clusters found. Although the analysis of variance when creating the signal is performed, the association distribution between clusters and behaviors is not homogeneous. For example, in a specific group of observed activities, 80% of the behaviors can be classified as habit 1, and the other 20% as close to cluster number two. In this case, habit 1 is the usual habit of the user, while habit 2 corresponds to unusual behaviors.

## 3. Correlation with the User Personality Trait

**Figure 4:**
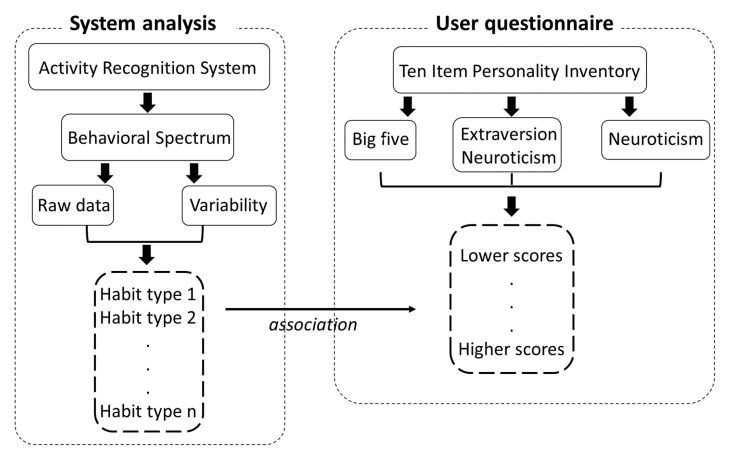
Methodology to find the correlation between habits and personality traits.

Patterns shown in the behavior signal can lead to identification of user habits, as mentioned in an earlier study [29]. However, instead of only identifying user habits, this work aimed to create an association between a few of the main habits of users and their personality trait scores, using several interpretations of the data as features. Additionally, because the approach to represent the sequence of user activities as the behavioral spectrum was proposed recently [29], it is not fully known yet how the other types of data extracted from the behavioral signal could be used to better understand user characteristics. Therefore, we used the methodology shown in Figure 4 to create these associations between user habits and personality trait scores.

### 3.1. System Analysis

First, two types of data were collected from a group of people. One is the activity information recorded during a specific period of time. Here, the sequence and duration for each activity are identified and collected. The activity information is expressed as one behavioral spectrum, as described before. The other type of data provided by the users comprises self-examination scores according to a personality trait questionnaire. The users will answer a list of questions to express their scores in all five dimensions according to the big five model. These two types of information (extroversion and neuroticism) were used to establish correlation between the personality traits of users and their habits and activities. Additionally, the neuroticism score was analyzed alone. The reason for performing this analysis is that the ROU model, which is closely related to human stability, was considered in this study. The resilient type has a score lower than the average on neuroticism, whereas the overcontrolled scores are lower than the average on extroversion and higher than the average on neuroticism. Furthermore, the undercontrolled one is high in both cases [7]. The neuroticism is also based on the activation thresholds in the sympathetic nervous system or visceral brain [37]. This is the part of the brain responsible for the fight-or-flight response in the face of danger. Activation can be measured based on the heart rate, blood pressure, cold hands, sweating tendency, and muscular tension (especially in the forehead) of individuals. Neurotic people, who have a low activation threshold, experience negative effects (fight-or-flight) in the face of very minor stressors; i.e., they are easily upset. Emotionally stable people, who have a high activation threshold, experience negative effects only in the face of very major stressors, and they are calm under pressure.

The data within the behavioral spectrum were then featured in two different ways. One way is to represent the raw data present in the signal obtained after Fourier transformation, which will be a vector having a length of 200 points. The second way to represent the behavioral spectrum is with its variability, which will be a combination of different features used to show the discrepancy present in the signal. The features used to express variability are the signal average, standard deviation, variation, auto correlation, and entropy. Using these values as variability indicators, we can represent two trials involving activities with the same durations but with different sequences. This division in the behavioral spectrum representation was performed to test different ways to use the activity information.

### 3.2. Comparison of User Questionnaire Scores

The scores obtained from the personality trait questionnaire were also featured in different ways. All the five dimensions were used, including extroversion and neuroticism dimensions, and then finally, only the neuroticism scores were used. This was done to observe the extent of correlation between the habits of the users and specific aspects of their personality traits. In other words, the objective of this division was to determine the correlation between a few specific dimensions and the user activities and habits. The output obtained from k-means is straightforward, and as such, the two groups into which the personality trait scores were classified represented all the scores obtained from the questionnaires. The first cluster obtained using k-means represented most of the users with low personality trait scores. The second cluster represented the high scores. However, this was not always true, particularly when all five dimensions were used as features; this is because users can have a high score on one dimension but a low score on another; therefore, their traits can be classified into a part of the first cluster.

After clustering all the features, each instance of the data was classified within the following two clusters; the first comprised habit type 1 and habit type 2, and the second comprised the low and high score groups in the questionnaire. If the habit group identified from the activity information matched the corresponding score group identified from the personality traits, we can say that there is an association between that trial or habit and the feature(s) selected as the personality trait(s). The association rate can be defined as the percentage of users associated with the same group after clustering the personality trait and activity features. Hence, this method provides a way of finding associations between a specific numerical representation of trials or habits of users and their personality traits, represented in the form of different features. For example, let us assume that one trial represented as raw data is classified as part of habit 1. However, the same trial represented as the variability extent is classified as part of habit 2. Then, we can determine the group into which the personality trait is classified using only the neuroticism as the input feature. If the output corresponds to the lower score group, we can conclude that raw data represented as a trial has higher correlations with personality traits than that observed when the raw data are represented using their variability. We performed the same procedure for all data instances in the available dataset and obtained the correlation distributions of all activity-related features and personality trait dimensions. Hence, to present a robust correlation between the selected features, it is necessary to have access to a larger dataset containing both the activity information required to determine the trials and personality trait scores; this aspect is described in the next subsection.

### 3.3. Dataset Description

**Figure 5:**
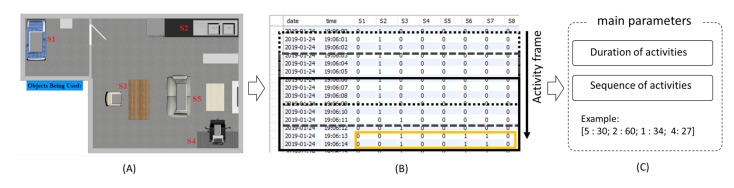
ALL data collection overview; (**A**) ALL environment layout. S1-S5 are each spaces, S1:bed room, S2:Kitchen, S3:dining room, S4:study room, S5:living room; (**B**) activity frame monitors the database and finds the activation values similar to the ones in the conditional matrix. The details of this process can be found in our previous work [20,33]; (**C**) The main parameters used in the personality trait assessment are the duration and sequence of activities.

To establish the proposed correlations proposed in this work, we had to use data collected from different users across different ages and personalities. Therefore, we used two different datasets in this work. This data collection was conducted in accordance with the Chubu University ethical guidance for research and the HS data collection has been approved by the institutional review board of JAIST, which is a research partner in the CARESSES project. The objective behind using two different datasets was to analyze the correlations between different ages and different time windows of the observed activities. The first dataset was obtained from previous works [29] (The overview of data collection is shown in Figure 5). Five different activities were chosen for observation in this set of data collections. The activities were cooking, eating, drinking, studying, sleeping, and watching TV. The data collection environment was designed in an area comprising a kitchen, dining room, bedroom, living room, and a study room. We conducted 29 trials using 24 subjects (*M*:21.71, *SD*:0.55) for two weeks, with AR experiment conducted for five days. The subjects were able to choose the activities to be executed in any desired order within a maximum time limit of 20 min. Several sensors were placed throughout the experimental area to automatically recognize the activities. The activity recognition system used was extracted from [20]. The system collected the data from each sensor in the network, and the activity frame parsed through the obtained activation values using a conditional matrix. The duration of activities and ID numbers of activities were extracted to identify the personality traits when the AR system acquired the user activities. More detailed information of the AR system can be found in our previous work [33]. The first set of experimental data obtained in this work will be referred to as ambient assisted living (AAL) data.

The second round of data collection was conducted in the HISUISUI (HISUISUI care home, hisui.or.jp), a care home for the elderly located in Ishikawa, Japan. Instead of setting a specific location and asking the users to accomplish the tasks, the daily activities of the subjects were recorded by therapists. No sensors were used for these collections. The behavioral spectrum could be conveniently obtained from the information recorded by therapists, because it included the duration and sequence of activities. All the identified activities were manually recorded by therapists. Twelve patients were observed (50% male), having an average age of 85.25 years (standard deviation 4.95). This set of data will be referred to as HS data.

Although it is difficult to validate these results and because extracting a specific personality trait is not an easy task, we used a 10-item personality measure (TIPI-J, adapted for Japanese users as the experiments were conducted in Japan) in the form of a questionnaire. The main objective of the TIPI questionnaire is to obtain scores related to each of the big five traits, namely extroversion, agreeableness, conscientiousness, neuroticism and openness, using 10 questions (i.e., two questions per personality score). For the subjects involved in the HS data experiments, the questionnaires were filled out by their therapists. The fact that the personality information was provided by the therapists could be a limitation of our study. It is therefore worthwhile to mention that the participants were elderly people living in care homes and some of them faced difficulties when answering the questionnaire, and therefore, assistance from the therapists was quite necessary.

## 4. Correlation Results between Personality Scores and Activity Series

### 4.1. Clustering Results

**Figure 6:**
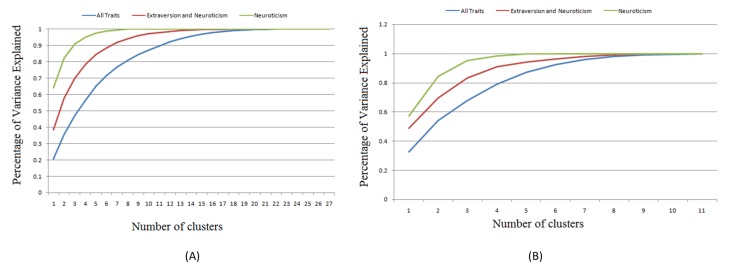
(**A**) Percentage of variance explained in the data based on the number of clusters in the AAL experiments using the personality scores as features, (**B**) Percentage of variance in the dataset explained based on the number of clusters employed for HS experiments using personality scores as features.

All of the above selected features were clustered separately using the k-means algorithm described in clustering behavioral spectrums to find user habits. To find the optimum number of clusters, or in other words the correct number of habits identified in a set of trials, the elbow method employed in a previous work was used [29]. This method calculates the percentage of the explained variation for each number of desired clusters. At one moment, as the number of clusters used in the k-means algorithm increases, there is no significant increment in the percentage of explained variation between the clusters and their respective indexes. The marginal point where the explained variation does not change substantially according to the number of points showing an apparent angle is marked in the elbow graph [38]. The results regarding the percentage of variation explained in the data obtained from the AAL experiments based on the number of clusters used are shown in Table 1, Figure 6A. The same analysis was performed using the HS data and the results are presented in Table 2. Figure 6B shows the HS results for the explained variations in the number of clusters using personality trait scores as features.

Comparing the percentage of explained variation obtained using both features to express activity information, we observed that using the variability yielded a higher percentage from a lower number of clusters compared to the behavioral spectrum represented in its raw format. Increasing the number of traits decreases the explained variation in the obtained clusters. Moreover, we observed a considerable difference when comparing the explained variation with the number of clusters according to the age of the subjects. For example, using five clusters for HS (elderly), we obtained a total explained variation of 85% (all traits); however, when using the same number of clusters for the 166A dataset (younger subjects, we obtained a total explained variation of 62% (all traits).

Comparing the obtained explained variance from the activity-related features from both AAL and HS data we inferred that using the variability obtained from the behavioral spectrum allowed us to obtain a higher explained variation for the same number of clusters. In addition, comparing the three different periods of the day showed that there is a higher possibility of users performing different activities during the morning period, which was true according to the annotation data.

Using the data provided by the behavioral spectrum can lead to situations where the percentage of variation does not change significantly on increasing the number of clusters. A higher percentage of variance was obtained with few clusters using only neuroticism as feature; the same result was obtained when using variability as well. This is explained considering that the user did not repeat the experiments during different days; instead, data were collected during only one instance per user. Therefore, we can state that almost all the users performed different trials. The results for all traits lead to a similar conclusion; it is difficult to cluster different users according to the scores of all their personality traits. However, when analyzing only neuroticism as a feature, it is possible to classify a larger number of users into the same group. This result shows that it is difficult to find the correct number of habits and clusters in the dataset. Therefore, in this work, both the behavioral spectrum and personality trait scores were clustered into two groups.

### 4.2. Personality Trait Identification Results

As explained in the Correlation with User Personality Traits section, two types of information were collected from the users, from two different datasets. The information related to the activity trials of the users were represented as behavioral signals. For this case, the features used were the behavioral signal in its raw format and the data variability found. Each of the features from each dataset was clustered into two groups. The clustering results for the personality scores showed that the output cluster number 1 represented the highest scores and the output cluster number 2 the lowest scores. The correlation is observed for the same user, and the activity trait is classified within the same cluster group with which the personality of the user is associated. Because users with higher levels of stability usually perform their activities in a slow and organized manner, all the routines fitting in the habits with higher amplitudes (the dotted red line) are associated with higher levels of stability in the N dimension based on the PEN or big five model. Higher levels of neuroticism are associated with the other two habits. All the routines found in the experiments were classified into one of the three routines; then, the behavior or user was associated with one personality trait.

To compare with psychological approach, we collected the TIPI-J as described in Section 3.3. The personality scores obtained from the questionnaire were organized in tree groups (all five scores, only extroversion and neuroticism dimensions, and only the neuroticism scores).

The durations of the trials observed in the activity data in the AAL experiments were shorter (maximum 20 min) than the ones observed in the HS data. Consequently, compared to longer observation times, the behavioral spectrum obtained from the AAL trials contained more activity information. Therefore, representation using the variability as a feature showed slightly higher associations with the user personality traits compared to that observed when using extroversion and neuroticism as features. However, when using all five personality traits, the raw data showed better association than that achieved using the variability feature (See Table 3). One possible conclusion from these results is that for a shorter period, the sequence and duration of the activities in a trial has a higher level of association with all five activity traits as a whole. In contrast, the variability in the user activity series has a higher level of association with extroversion and neuroticism scores. Furthermore, there is no significant association between the activities and neuroticism alone.

The activities collected from the HS dataset were divided in three periods of the day, namely morning, afternoon, and night (See Table 4, Table 5 and Table 6). This classification was done to observe the influence of each group of activities on the association, because the elderly normally have a distinct separation in terms of the activity frequency according to the period of the day. The results for the association observed during the morning period are presented in Table 4.

For the HS experiments, the results showed that the association between all the big five scores and activity features as behavioral signals was high. One reason for this is that the scores obtained from the elderly in terms of extroversion and neuroticism did not change much between the individuals, or at least the therapists did not report any such changes. During the morning period, the number of observed activities was higher; however, the variability feature does not have a higher correlation than the behavioral spectrum in its raw format. One reason for this is that the variability in the number of activities between the individuals does not change the difference in the personality scores observed. This became clearer when observing the results for the night period.

The activities performed after lunch and before 6pm were considered as happening during the afternoon period. Their associations with the personality scores are presented in Table 5.

The trials observed for the afternoon period were almost the same for all the subjects. During that period, as part of rehabilitation and therapy, normally the subjects were encouraged to engage in group activities. This resulted in trials involving similar activities. Only a few subjects, owing to physical limitations, performed different activities. Furthermore, as stated before, because there is no significant difference in the personality trait scores, a high association between the activities and personality traits of the users was observed for the afternoon period. Finally, the association results obtained for the night period are presented in Table 6.

After analyzing the recorded trials for the night period, we found a moderate variability in the frequency and number of activities. Usually, older subjects tend to sleep very early, and do not perform any of the night activities, like watching TV or playing games. For this period, we found that neuroticism had a higher association with the activities in both featured representations (raw data and variability). In addition, the higher association found for the behavioral spectrum in its raw format to all the five traits was most likely because of the same reason mentioned for the association with the morning results explained earlier. The number and frequency of activities performed by the subjects were not consistent with the different personality traits. However, for the night period, the activities were not as diverse as the ones found in the morning, thus resulting in a lower level of association than that observed in the morning results for the five scores.

## 5. Discussion and Conclusions

In this work, we determined associations between user trials (comprising a sequence of activities) and their personality scores. Both the correlated aspects were features expressed in different ways to obtain a better understanding regarding how the frequency and number of activities are related to different user personality traits.

The result showed that for both young and elderly subjects, there is an association between the user personality traits and the manner in which they perform their activities. The association depends on how diverse the user personality traits are, when compared to the number of accomplished activities in a period of time. The associations for the young subjects tended were mostly found when using the extroversion and neuroticism features. For this particular case, an appropriate representation of user activities can be provided by analyzing their variability values. The above statement is supported by the results obtained using data collected from an elderly group by analyzing their activities for a longer time period. In the period of the day where a high number of activities was performed, the number of activities was not well correlated with specific traits; this is because the personality trait scores obtained from the subjects in this specific case are not as diverse as the ones found for the young subjects. For a group of subjects where the personality traits do not vary much, there is an association in smaller trials, using both the features to represent the sequence of activities.

Because our approach of using Behavioral Spectrum as explained in Section 2 is novel with regard to the representation of human habit, we also calculated the variability to see the effect of parameters in the numerical representation signal. The results presented in Table 4 indicate the significant association rates of neuroticism in behavioral spectrum variability (p = 0.017). The HS night period in (Table 6) shows high association raw data and variability (p = 0.017). This approach of estimating the personality traits of users helped us in identifying a numerical approach for quantification analysis, however, we restrict the conclusive statements regarding the personality features. As noted by the result presented in Table 3, although the associate rates in the extroversion and neuroticism variability is slightly higher, both the raw data and variability did not indicate significant associations. In Table 5 no statistically significant relationships are observed in any aspect of the personality traits. This can be attributed to the limited number of activities. Because the dataset contained various users’ activities performed in a day, the result could not reflect sufficient association with the personality traits. However, none of the methods were able to assess the personality traits based on simple sensor-based activity information. Therefore, future work can focus on addressing the aforementioned issues. First, the dataset used in this study can be expanded to a larger number of subjects and longer activity periods. Second, as noted by Donnellan el al. [7,8] ROU classification that focuses on neuroticism can be included to analyze user personality traits based on their activities.

The findings presented in this work showed the potential of the behavioral spectrum as a feature to represent a sequence of activities. Such information can be helpful for several human interaction applications for assessing the activity of the users as well to change the interaction degree according to those activities. Knowledge regarding the association between the frequency of the observed activities and user personality traits can improve interactive experiences when using smart home applications and domestic robots.

## Figures and Tables

**Table 1:** Percentage of variance explained based on the number of clusters in AAL data obtained using personality trait scores.

ALL Dataset			
Number of Clusters	Variance Explained (%)		
	All Five Traits	Extroversion Neuroticism	Neuroticism
1	0.2056	0.383271	0.640277
2	0.470641	0.578762	0.821736
3	0.567452	0.700684	0.911073
4	0.649912	0.784866	0.951275
5	0.715607	0.845282	0.975167
6	0.769358	0.88677	0.987584
7	0.809432	0.920082	0.995973
8	0.844376	0.942652	1
9	0.873644	0.959033	1
10	0.899276	0.971873	1
11	0.92267	0.980246	1
12	0.941895	0.986584	1

**Table 2:** Percentage of variance explained in the data based on the number of clusters in the HS data obtained using personality trait scores.

HS Dataset			
No. of Clusters	Variance Explained		
	All Traits	Extroversion Neuroticism	Neuroticism
1	0.326387	0.488978	0.573366
2	0.540396	0.697463	0.844636
3	0.679401	0.833466	0.95561
4	0.79117	0.910873	0.985203
5	0.872182	0.943198	1
6	0.92555	0.965009	1
7	0.960495	0.981561	1
8	0.981702	0.992478	1
9	0.991811	0.998868	1
10	0.997622	1	1

**Table 3:** Association between personality traits and user activity trials in AAL data.

Personality Types	Association Rate; Correlation Coefficient	
Personality trait features	Behavioral Spectrum raw data	Behavioral Spectrum variability
All five traits	0.64; 0.34 (p = 0.072)	0.60; 0.34 (p = 0.072)
Extroversion and Neuroticism	0.57; 0.34 (p = 0.072)	0.67; 0.34 (p = 0.072)
Neuroticism	0.53; 0.25 (p = 0.184)	0.53; 0.25 (p = 0.184)

**Table 4:** Association between personality traits and user activity trials in HS data obtained during the morning period.

Personality Types	Association Rate; Correlation Coefficient	
Personality trait features	Behavioral Spectrum raw data	Behavioral Spectrum variability
All five traits	0.75; 0.25 (p = 0.433)	0.58; 0.35 (p = 0.259)
Extroversion and Neuroticism	0.58; 0.47 (p = 0.115)	0.58; 0.17 (p = 0.599)
Neuroticism	0.50; 0.44 (p = 0.144)	0.50; 0.66 (p = 0.017)

**Table 5:** Association between personality traits and user activity trials in HS data obtained during the afternoon period.

Personality Types	Association Rate; Correlation Coefficient	
Personality trait features	Behavioral Spectrum raw data	Behavioral Spectrum variability
All five traits	0.83; 0.42 (p = 0.166)	0.83; 0.42 (p = 0.166)
Extroversion and Neuroticism	0.50; 0.35 (p = 0.254)	0.50; 0.35 (p = 0.254)
Neuroticism	0.58; 0.30 (p = 0.340)	0.58; 0.30 (p = 0.340)

**Table 6:** Association between personality traits and user activity trials in HS data during the night period.

Personality Types	Association Rate; Correlation Coefficient	
Personality trait features	Behavioral Spectrum raw data	Behavioral Spectrum variability
All five traits	0.66; 0.25 (p = 0.433)	0.58; 0.23 (p = 0.454)
extroversion and Neuroticism	0.66; 0.48 (p = 0.107)	0.66; 0.31 (p = 0.319)
Neuroticism	0.83; 0.66 (p = 0.017)	0.83; 0.66 (p = 0.017)
